# Nonleisure-time physical activity as a protective factor against sarcopenia in hemodialysis patients: a prospective cohort study

**DOI:** 10.3389/fnut.2025.1517429

**Published:** 2025-03-26

**Authors:** Liyang Chang, Yujuan Zheng, Yan Ding, Zhiqiao Long, Hongmei Zhang

**Affiliations:** Department of Renal Centre, Hangzhou TCM Hospital Affiliated to Zhejiang Chinese Medical University, Hangzhou, China

**Keywords:** maintenance hemodialysis, sarcopenia, physical activity, nonleisure-time physical activity, leisure-time physical activity

## Abstract

**Objectives:**

Sarcopenia is prevalent among individuals undergoing maintenance hemodialysis (MHD) and is influenced by sedentary lifestyles. Although leisure-time physical activities have been shown to prevent sarcopenia in patients undergoing MHD, the impact of nonleisure-time physical activities on sarcopenia has not yet been examined in prospective studies.

**Methods:**

This prospective cohort study, conducted in 2020 with a 12-month follow-up, included stable MHD patients without baseline sarcopenia. Sarcopenia was diagnosed according to the 2019 Asian Working Group for Sarcopenia criteria. Physical activity was assessed using the International Physical Activity Questionnaire. Additionally, demographic, dietary, nutritional, and laboratory data were collected. Modified Poisson regression analysis was employed to evaluate the impact of physical activity on the risk of developing sarcopenia.

**Results:**

Among the 196 MHD patients who completed the 1-year follow-up, 29 (14.8%) developed sarcopenia. The average total physical activity was 1,268 METs/week, with leisure-time activity averaging 300 METs/week and nonleisure-time activity averaging 724 METs/week. Adjusted analyses indicate that leisure-time physical activities do not significantly affect the risk of sarcopenia (RR = 0.920, 95% CI = 0.477–1.951; P > 0.05), whereas nonleisure-time physical activities are significantly associated with a reduced risk of sarcopenia (RR = 0.449, 95% CI = 0.248–0.814).

**Conclusion:**

Actively participating in physical activities (nonleisure-time physical activities) can reduce the incidence of sarcopenia in patients undergoing MHD. Promoting such activities may be an effective strategy to enhance physical fitness and mitigate sarcopenia risk among this population.

## 1 Introduction

Chronic kidney disease (CKD) has become a global public health issue, with a high prevalence rate of 9.1%, characterized by a progressive loss of kidney function (1). Maintenance hemodialysis (MHD) is a critical treatment for sustaining the lives of patients with CKD, accounting for 91% of all dialysis treatments (2). MHD replaces kidney function, enabling survival despite severe renal impairment. However, long-term MHD dependence leads to complications that affect health and quality of life (3, 4).

Sarcopenia, characterized by the progressive loss of skeletal muscle mass (SMM) and strength (5), is highly prevalent among patients on MHD, with rates ranging from 13.5% to 73.5% (6–9). Sarcopenia significantly impairs patients' physical functioning and independence, making it a critical concern in the management of CKD patients undergoing MHD. Furthermore, sarcopenia is associated with adverse clinical outcomes, including disability, reduced quality of life, and increased morbidity and mortality (9, 10). These outcomes not only affect patients' wellbeing but also impose substantial economic burdens on healthcare systems due to increased medical interventions and prolonged hospital stays (9). Several complex factors contribute to the development of sarcopenia in patients with MHD, including protein–energy wasting, hormonal imbalance, comorbidities, acid metabolism disturbance, inflammation, and a general lack of physical activity (11).

Physical activity encompasses any bodily movement produced by skeletal muscles that results in energy expenditure (12). It is categorized into leisure-time activities (such as aerobic and anaerobic exercises) and nonleisure-time activities (including household, occupational, and transport-related activities) (13). Physical activity may alleviate sarcopenia in patients undergoing MHD through various biological mechanisms, such as stimulating muscle protein synthesis, enhancing mitochondrial function, improving insulin sensitivity, reducing chronic inflammation, and supporting muscle regeneration (14, 15). While previous research has established a strong association between physical activity and sarcopenia (16, 17), most of the evidence focuses on leisure-time physical activities. In contrast, the clinical impact of non-leisure-time physical activity, which constitutes a significant portion of daily physical activity (18–22), remains underexplored, especially in populations with chronic diseases.

Additionally, patients on MHD often face challenges in participating in leisure-time physical activities due to dialysis-related weakness and fatigue, dialysis braking, concerns, and a lack of knowledge about such activities (23, 24). Decreased physical activity can trigger skeletal muscle atrophy, which further discourages patients on MHD from engaging in physical activities (25, 26). This creates a vicious cycle that ultimately exacerbates sarcopenia (25–27). Conversely, MHD patients may find it easier to adopt lifestyle-oriented nonleisure-time physical activities, thereby improving their physical activity levels, quality of life, and compliance (28, 29).

Therefore, this prospective cohort study aims to explore the relationship between various types of physical activity and the risk of sarcopenia in patients undergoing MHD. We hypothesize that increasing nonleisure-time physical activity levels may reduce the risk of sarcopenia, similar to leisure-time physical activity, in this population.

## 2 Methods

### 2.1 Study design and participants

This 1-year prospective cohort study builds on Ding et al.'s cross-sectional research on sarcopenia in patients undergoing MHD (8). Conducted from September 2020 to January 2022 at Hangzhou TCM Hospital, Zhejiang Chinese Medical University, China, the study included participants who were: (1) free of sarcopenia as per the Asian Working Group for Sarcopenia (AWGS); (2) age ≥18 years; (3) on hemodialysis for at least 3 months, undergoing dialysis three times weekly; and (4) capable of independent mobility. Exclusion criteria were: (1) trauma, severe infections, or malignant tumors within the past 3 months; (2) cognitive impairments; and (3) refusal to participate in the study or withdrawal during the study due to reasons such as lack of interest, time constraints, or limitations related to physical health.

### 2.2 Sarcopenia diagnosis and measurements

According to the diagnostic consensus of the AWGS (30), patients who meet the following criteria (1) and (2) and/or (3) are diagnosed with sarcopenia: (1) muscle mass loss defined as skeletal muscle index (SMI) < 7.00 kg/m^2^ for men and < 5.70 kg/m^2^ for women; (2) muscle strength reduction defined as handgrip strength (HGS) < 28 kg for men and < 18 kg for women; and (3) muscle dysfunction defined as 6-m gait speed (GS) < 1 m/s.

#### 2.2.1 Bioimpedance measurements

Body composition was assessed using the Seca515 dual-energy electrical impedance analyzer (Seca GmbH & Co., Hamburg, Germany) 30 min post-dialysis, following bioelectrical impedance analysis (BIA) guidelines (31). Patients fasted for 2 h, emptied their bladders, removed metallic objects, wore lightweight clothing of known weight, and stood barefoot on the device. They held the handles with both hands, extended their arms, and entered personal information [name, age, sex, height, waist circumference (WC)] into the system. The Seca515 analyzer utilizes eight-electrode technology to perform segmented impedance measurements at a 100-volt current, relying on the device's proprietary algorithms for all calculations. SMM measurements were recorded at a frequency of 50 kHz, with results displayed in kilograms. Formulas used:

SMI (kg/m^2^) = SMM (kg)/height^2^ (m^2^) (32).

Body mass index (BMI): BMI (kg/m^2^) = weight (kg)/height^2^ (m^2^).

#### 2.2.2 Muscle strength and function measurements

Prior to dialysis (after a short no-dialysis pause of one day), HGS was measured using a custom electronic meter (Guangdong Xiangshan Weighing Apparatus Group, China). Patients stood upright using their nonfistulated or dominant hand, fully extended elbow, exerted maximum force, rested for 3 min, and repeated the measurement. The higher of the two HGS values (kg) was recorded. GS was evaluated by having patients walk 6 m at their usual pace without assistance. After a 3-min rest, GS was measured again, and the average of the two GS values (m/s) was used (33). All measurements were conducted by the same evaluator during the same dialysis session as BIA.

### 2.3 Parameters of demographic and laboratory

Demographic and laboratory data were extracted from the electronic medical records, selecting routine fasting pre-dialysis laboratory test results within 4 weeks before and after the measurement. Variables included age, sex, duration of hemodialysis (dialytic age), primary disease, education level, comorbidities, blood urea nitrogen (BUN), serum calcium (Ca^2+^), glucose (GLU), intact parathyroid hormone (iPTH), phosphorus (P^3−^), total cholesterol (TCH), high-sensitivity C-reactive protein (hs-CRP), albumin (Alb), and hemoglobin (Hb).

### 2.4 Diet and nutritional status measurements

Dietary protein intake was estimated using the normalized protein equivalent of nitrogen appearance (nPNA) (34). Nutritional status was assessed with the Modified Quantitative Subjective Global Assessment (MQSGA) (35–37), evaluating weight changes, dietary intake, gastrointestinal symptoms, functional status, dialysis complications, subcutaneous fat loss, and muscle wasting over the past 6 months. Each category was scored from 1 (normal) to 5 (severe malnutrition), with total scores ranging from 7 to 35. Scores of 7–10 indicate normal nutrition, 11–20 indicate mild to moderate malnutrition, and 21–35 indicate severe malnutrition.

### 2.5 Physical activity evaluation

Physical activity levels were assessed using the International Physical Activity Questionnaire (IPAQ), a reliable and valid tool (38–40). The IPAQ measures nonleisure-time activities (occupation, housework, transportation) and leisure-time activities (aerobic, anaerobic) over the past week, quantified in metabolic equivalents (METs). Total energy expenditure was calculated based on the frequency, duration, and intensity of activities during seven consecutive days.

Participants were categorized into active or inactive groups according to the American College of Sports Medicine guidelines (41). The active group met at least one of the following criteria: (1) Vigorous-intensity activities ≥3 days/week, ≥20 min/day; (2) Moderate-intensity or walking activities ≥5 days/week, ≥30 min/day; (3) Any combination achieving ≥600 MET min/week. Participants not meeting these criteria were classified as inactive.

### 2.6 Management and follow-up

Participants underwent face-to-face assessments conducted by a single evaluator to ensure consistency. Biochemical indicators were measured at baseline, with laboratory tests performed in the early morning under fasting conditions using the automated biochemical analyzer (Mindray BS800) on samples from internal fistulas or catheters. Nutritional status, SMM, HGS, and GS were assessed at baseline and after one year. Physical activity was measured twice shortly after enrollment (weekly) and then monthly during the follow-up, with analysis based on the average of the first two IPAQ results. During the follow-up period, patients continued routine MHD treatments, were encouraged to maintain a healthy lifestyle, and did not receive additional interventions for preventing or treating sarcopenia.

### 2.7 Statistical analysis

Data were analyzed using IBM SPSS Statistics version 25.0 (IBM Corp., Armonk, NY, USA). Normally distributed variables are presented as mean ± standard deviation (SD), non-normally distributed variables as median (interquartile range, IQR), and categorical data as frequencies and percentages. Between-group comparisons for continuous variables used independent *t*-tests, chi-square tests for categorical variables, and Mann–Whitney U-tests for non-normal data. Sparse Principal Component Analysis (SPCA) was used for dimensionality reduction and feature extraction. A bi-plot was generated to visualize variable loadings and patient distribution. Pearson's correlation analysis was performed to evaluate the relationships between variables. A correlation heatmap was generated to visually represent the strength and direction of the associations. Using modified Poisson regression analysis to explore the relationship between physical activity and the risk of sarcopenia. Using METs on the x-axis and relative risk on the y-axis to illustrate the dose-response relationship between METs of physical activity and the risk of sarcopenia. A two-sided P < 0.05 was considered statistically significant.

## 3 Results

### 3.1 Participant characteristics

**Figure 1:**
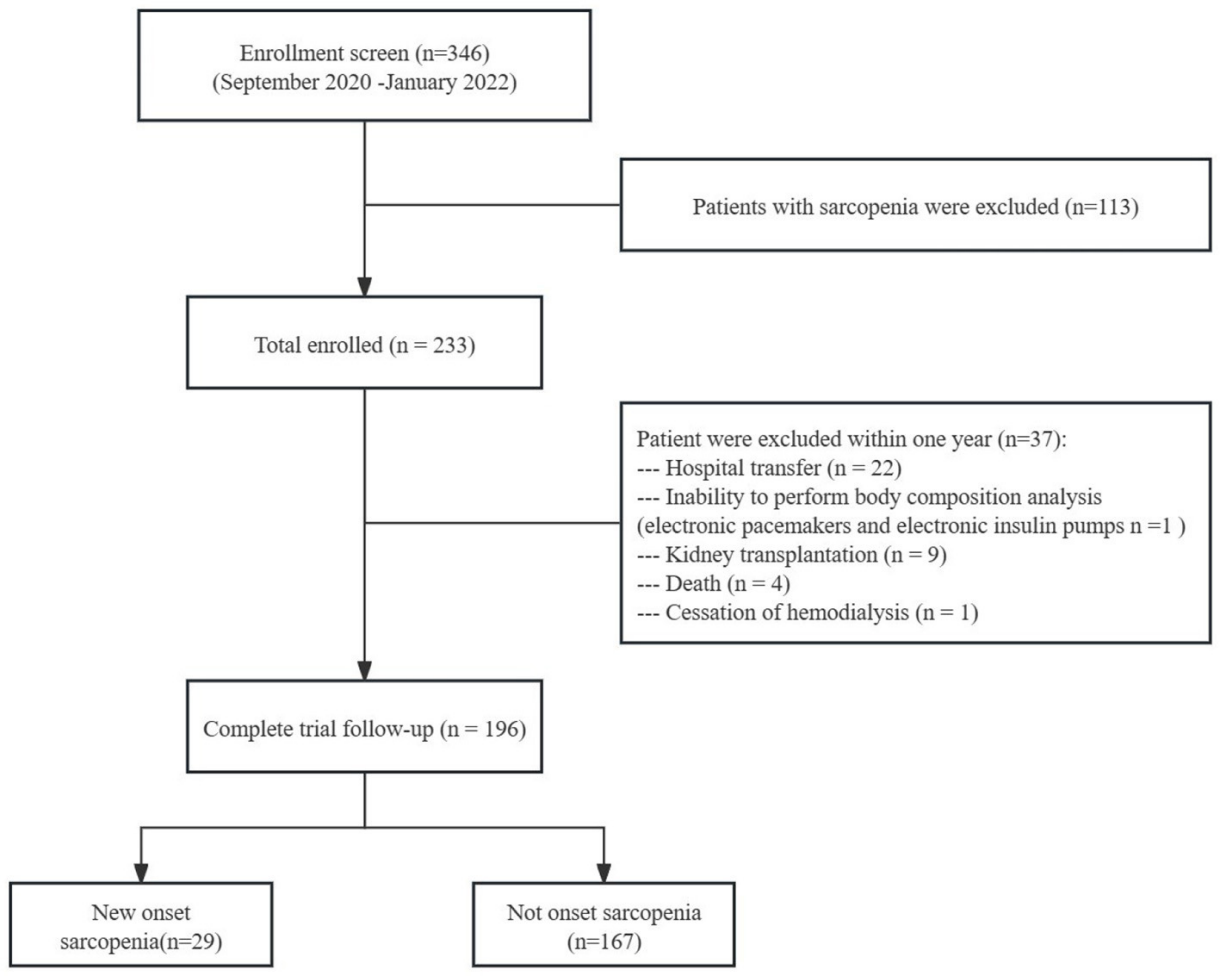
Flow chart of the study participants.

Of the 346 patients screened, 233 without sarcopenia were initially included (8). After excluding 37 patients due to hospital transfer, kidney transplantation, death, inability to perform body composition analysis, or cessation of hemodialysis within 1 year, 196 patients completed the study. Among them, 29 (14.8%) were diagnosed with sarcopenia (Figure 1). Baseline characteristics of the sarcopenia and nonsarcopenia groups are summarized in Table 1. Significant differences between the groups were found in age, sex, ALB, BMI, MQSGA, WC, HGS, and SMM (*P* < 0.05).

**Table 1:** General information of the sarcopenia and nonsarcopenia groups.

**Parameters**	**Total**	**Sarcopenia**	**Nonsarcopenia**	***P***
	**(*****n*** = **196)**	**(*****n*** = **29)**	**(*****n*** = **167)**	
Age (y)	53.57 ± 12.43	60.34 ± 10.35	52.40 ± 12.41	0.001
**Sex**, ***n*** **(%)**				0.003
Men	131 (66.8%)	12 (41.4%)	119 (71.3%)	
Women	65 (33.2%)	17 (58.6%)	48 (28.7%)	
Dialysis vintage (mo)	52 (21.25,110)	97 (24,146.5)	47 (21,105)	0.148
**Protopathy**, ***n*** **(%)**				0.238
Diabetic nephropathy	46 (23.5%)	4 (13.8%)	42 (25.1%)	
Others	150 (76.5%)	25 (86.2%)	125 (74.9%)	
**Education level**, ***n*** **(%)**				0.422
≤ Middle school	99 (50.5%)	17 (58.6%)	82 (49.1%)	
≥High school	97 (49.5%)	12 (41.4%)	85 (50.9%)	
BUN (mmol/L)	22.19 ± 5.27	20.88 ± 4.38	22.42 ± 5.39	0.146
TCH (mmol/L)	3.85 (3.29, 4.53)	4.12 (3.45, 4.75)	3.82 (3.29, 4.49)	0.287
hs-CRP (g/L)	2.20 (1.12, 4.53)	1.90 (1.24, 3.25)	2.21 (1.06, 4.89)	0.419
P^3−^ (mmol/L)	1.85 (1.58, 2.19)	1.92 (1.64, 2.1)	1.84 (1.58, 2.22)	0.976
Ca^2+^ (mmol/L)	2.32 (2.19, 2.42)	2.33 (2.2, 2.43)	2.32 (2.19, 2.4)	0.696
ipTH (pg/ml)	329.85 (197.88, 560.48)	435.1 (219.65, 553.8)	325.0 (187.8, 565.1)	0.504
GLU (mmol/L)	5.84 (4.72, 7.92)	6.57 (4.70, 7.98)	5.59 (4.71, 7.78)	0.836
HB (g/L)	113.34 ± 14.78	113.17 ± 14.55	113.37 ± 14.86	0.948
ALB (g/L)	39.6 (38.1, 41.58)	38.7 (36.85, 40.45)	39.8 (38.2, 42.1)	0.009
BMI (kg/m^2^)	23.43 ± 3.69	20.25 ± 2.51	23.98 ± 3.59	0.001
MQSGA (score)	10 (10, 11.75)	12 (10, 12)	10 (9, 10)	0.001
nPNA (g/kg/d)	1.10 ± 0.24	1.10 ± 0.22	1.10 ± 0.24	0.972
WC (cm)	0.86 ± 0.12	0.79 ± 0.11	0.87 ± 0.12	0.001
Sedentary time (hours/week)	56 (42, 56)	56 (42, 56)	56 (42, 56)	0.907
HGS (kg)	31.06 ± 9.30	23.03 ± 6.90	32.45 ± 8.96	0.001
SMM (kg)	21.90 ± 4.84	17.12 ± 2.91	22.73 ± 4.63	0.001

ALB, albumin; BMI, body mass index; BUN, blood urea nitrogen; Ca^2+^, serum calcium; GLU, glucose; HB, hemoglobin; HGS, handgrip strength; hs-CRP, high-sensitivity C-reactive protein; ipTH, parathyroid hormone; MQSGA, modified quantitative subjective global assessment; nPNA, normalized protein equivalent of nitrogen appearance; P^3−^, serum phosphorus; SMM, skeletal muscle mass; TCH, total cholesterol; WC, waist circumference. Two independent-sample t-test, Mann–Whitney U-test, and chi-square test were used in normally distributed quantitative data, non-normally distributed quantitative data, and qualitative data, respectively.

### 3.2 Physical activity evaluation

**Figure 2:**
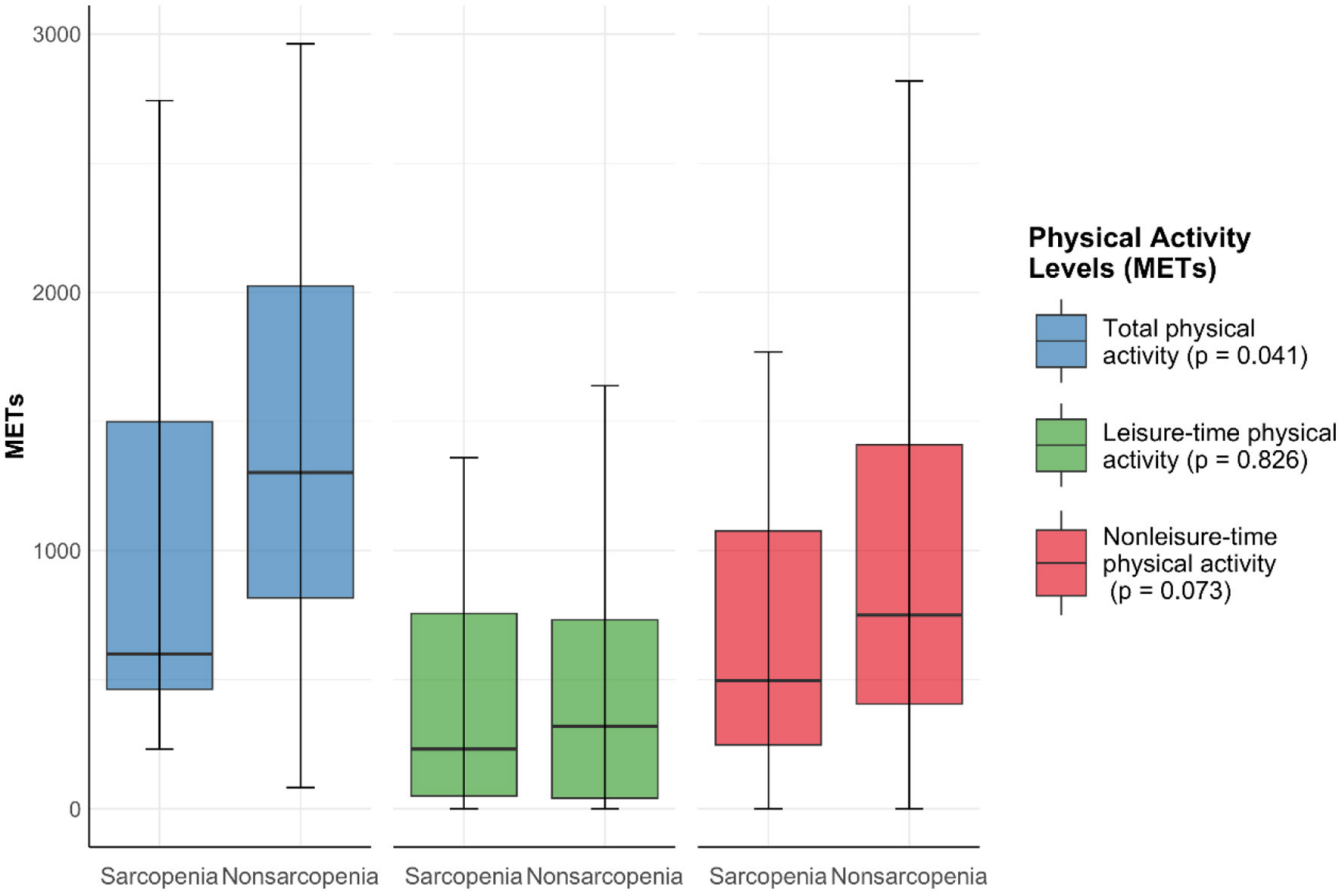
Physical activity levels between sarcopenia group and nonsarcopenia group. Data expressed as median ± range.

The total physical activity was 1,268 (687, 1,981) METs/week. The leisure-time and nonleisure-time physical activities were 300 (37, 740) and 724 (369, 1,338) METs/week, respectively. Nonsarcopenia patients exhibit higher physical activity levels than sarcopenia patients across all categories measured. They are more active in total physical activity (*P* = 0.041), leisure-time physical activity (*P* = 0.826), and nonleisure time physical activity (*P* = 0.073). However, there was no statistically significant difference in leisure-time physical activity and nonleisure-time physical activity between the two groups (Figure 2).

### 3.3 Sparse principal component analysis of sarcopenia-related factors

**Figure 3:**
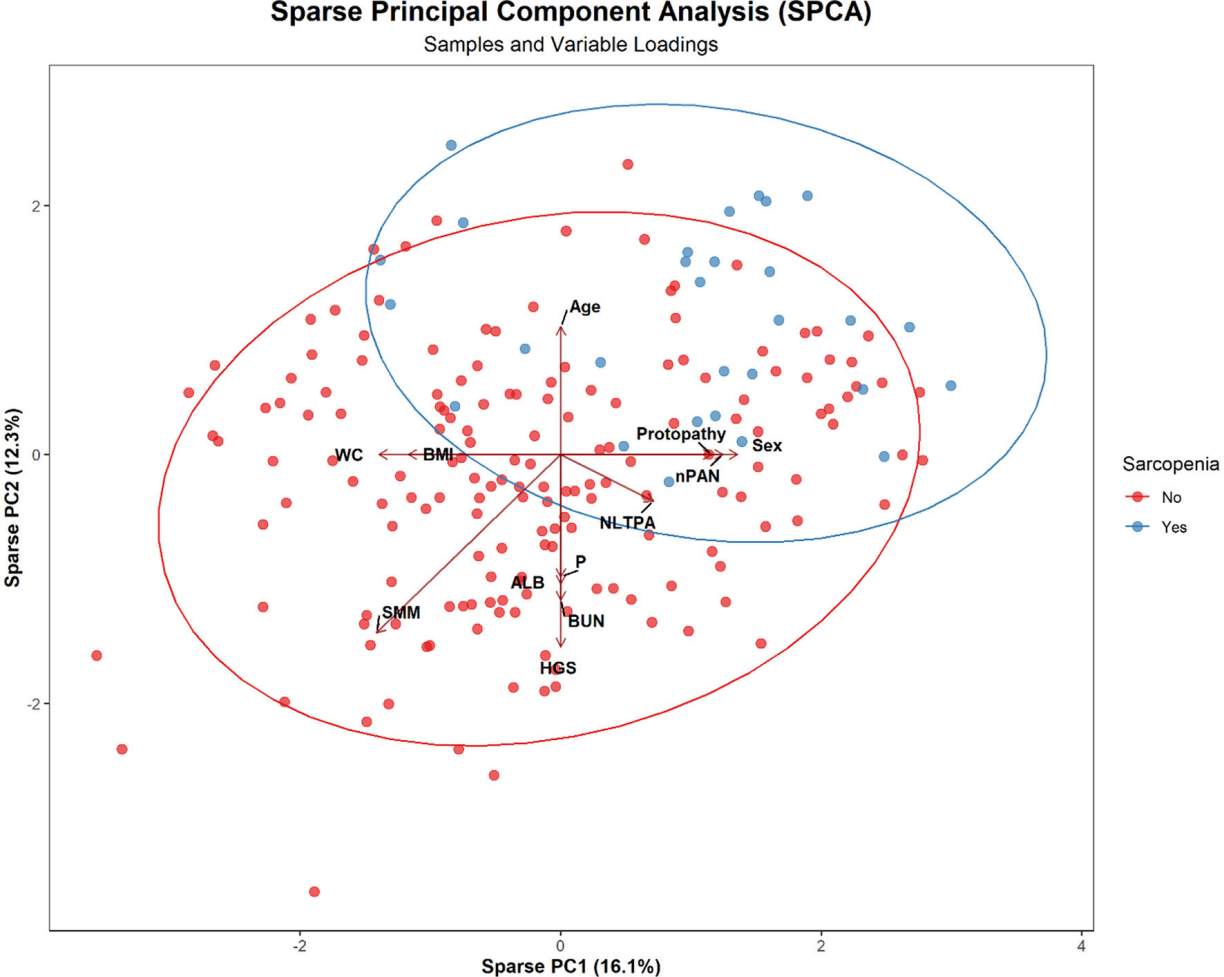
SPCA Bi-plot showing the distribution of sarcopenia and non-sarcopenia patients with key variable loadings.

Figure 3 presents the SPCA bi-lot, where blue and red dots represent individuals with and without sarcopenia, respectively, and ellipses indicate the 95% confidence interval. The first two principal components (PC1: 16.1%, PC2: 12.3%) explain 28.4% of the total variance. Nonleisure-time physical activity, HGS, SMM, BMI, WC, and ALB align with PC1, suggesting their importance in distinguishing sarcopenia status. Age, Protopathy, nPAN, and Sex align with PC2, indicating their influence on individual distribution. Sarcopenia cases cluster in the upper-right region, associated with Age and disease-related factors, while non-sarcopenia cases concentrate in the lower-left, linked to higher muscle mass and nonleisure-time physical activity.

### 3.4 Relationship between physical activity types and sarcopenia risk

**Figure 4:**
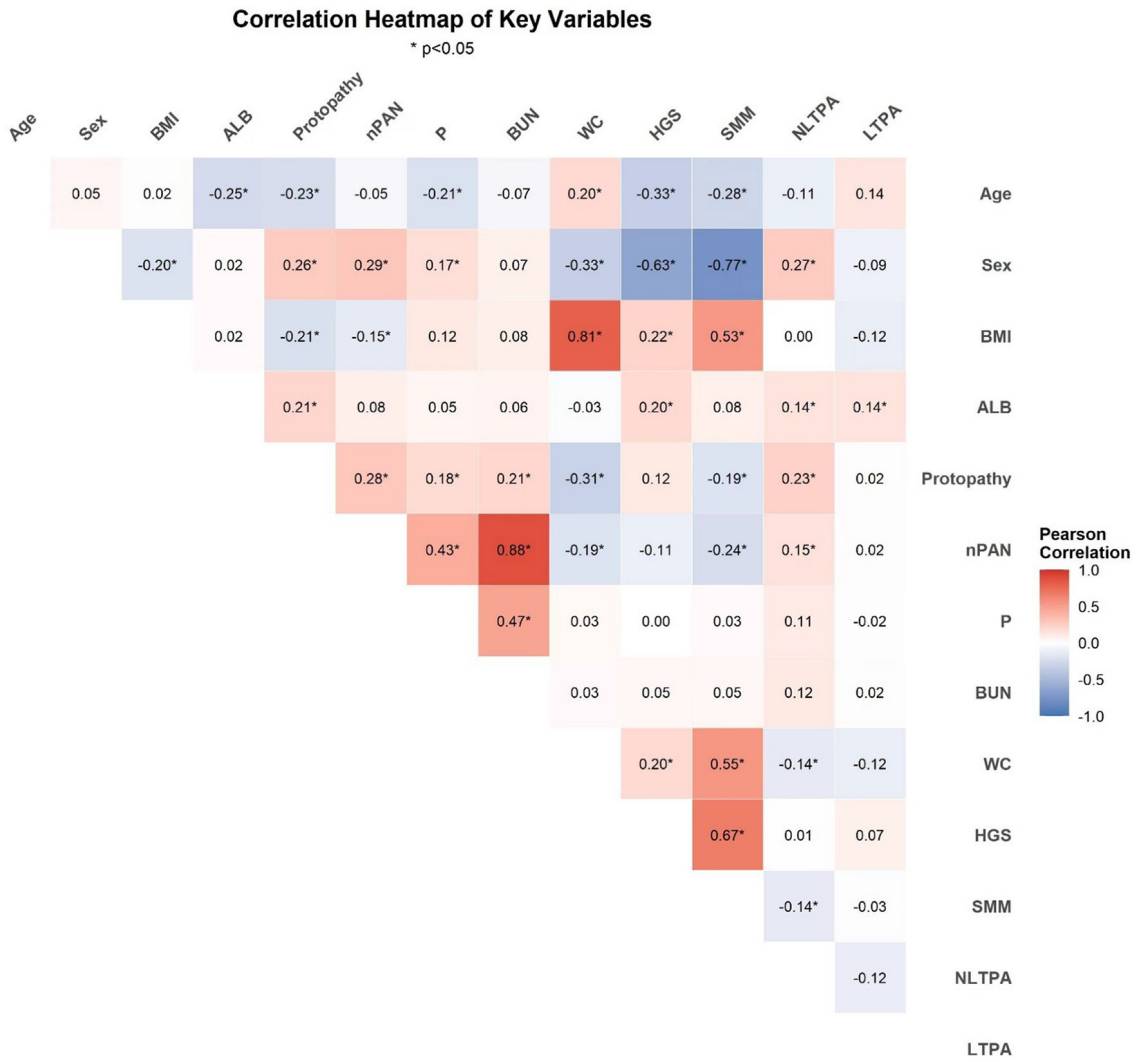
Correlation heatmap of physical activity, demographics, nutritional indicators, biochemical markers, and muscle mass parameters in MHD patients (*P < 0.05).

**Figure 5:**
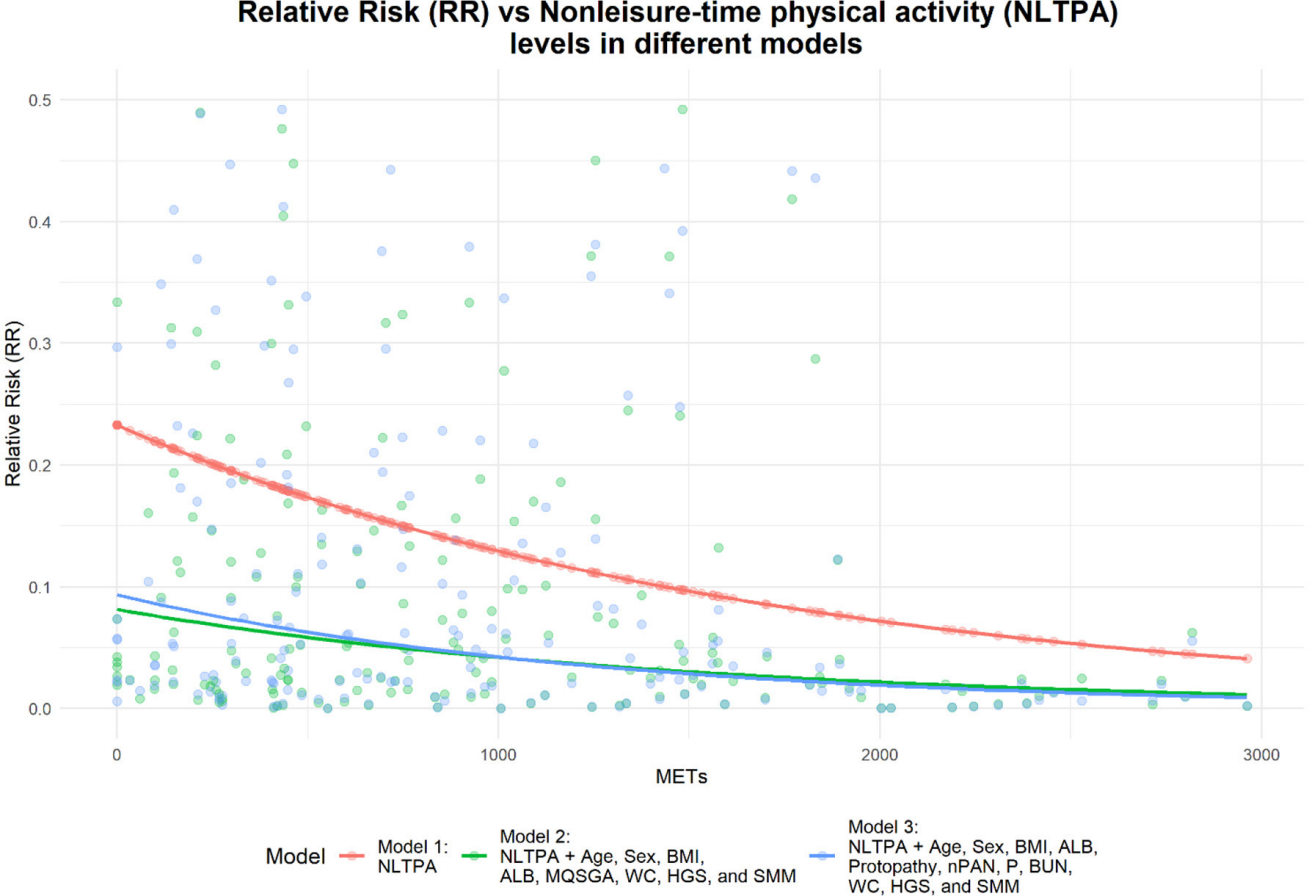
Relationship between nonleisure-time physical activity levels and sarcopenia risk.

Among 196 adults, 67 (34.2%) and 112 (57.1%) actively participated in leisure-time and nonleisure-time physical activities, respectively (Table 2). Figure 4 presents a correlation heatmap illustrating the relationships between physical activity, demographic characteristics, nutritional indicators, biochemical markers, and muscle mass parameters in MHD patients. Using the inactive group as the control group for comparison, after adjusting for age, sex, BMI, ALB, MQSGA, WC, HGS, and SMM, active participation in both leisure-time and nonleisure-time physical activities negatively correlated with sarcopenia risk; however, only nonleisure-time physical activity was significantly associated with sarcopenia risk (*P* < 0.05). After further adjustment for age, sex, BMI, ALB, protopathy, nPAN, P, BUN, WC, HGS, and SMM, nonleisure-time physical activity was still negatively correlated with sarcopenia risk (nonleisure-time physical activity: RR = 0.449, 95% CI = 0.248–0.814, *P* < 0.05) (Table 3). Using nonleisure-time physical activity level as the X-axis and RR as the Y-axis, RR shows a decreasing trend as nonleisure-time physical activity increases (Figure 5).

**Table 2:** Characteristics of the participants in the LTPA and NLTPA groups.

**Parameters**	**LTPA**			**NLTPA**		
	**Inactive**	**Active**	***P***	**Inactive**	**Active**	***P***
	**(*****n*** = **129)**	**(*****n*** = **67)**		**(*****n*** = **84)**	**(*****n*** = **112)**	
Age (y)	53.0 ± 12.76	54.67 ± 11.78	0.373	54.63 ± 12.09	52.78 ± 12.67	0.303
**Sex**, ***n*** **(%)**			0.751			0.001
Men	85 (65.9%)	46 (68.7%)		68 (81.0%)	63 (56.3%)	
Women	44 (34.1%)	21 (31.3%)		16 (19.0%)	49 (43.8%)	
Dialysis vintage (mo)	49 (21.5, 121.5)	56 (21, 105)	0.987	38 (16, 118.5)	56 (26.5, 107.75)	0.159
**Protopathy**, ***n*** **(%)**			0.216			0.017
Diabetic nephropathy	34 (26.4%)	12 (17.9%)		27 (32.1%)	19 (17.0%)	
Others	95 (73.6%)	55 (82.1%)		57 (67.9%)	93 (83.0%)	
**Educational level**, ***n*** **(%)**			0.765			0.043
≤ Middle school	64 (49.6%)	35 (52.2%)		35 (41.7%)	64 (57.1%)	
≥High school	65 (50.4%)	32 (47.8%)		49 (58.3%)	48 (42.9%)	
BUN (mmol/L)	21.81 ± 5.21	22.93 ± 5.34	0.156	22.22 ± 5.54	22.17 ± 5.08	0.946
TCH (mmol/L)	3.85 (3.30, 4.50)	3.9 (3.29, 4.66)	0.728	3.80 (3.31, 4.38)	3.91 (3.27, 4.67)	0.354
hs-CRP (g/L)	2.34 (1.17, 4.50)	1.83 (0.98, 4.89)	0.305	2.49 (1.23, 5.34)	2.00 (0.99, 4.15)	0.265
P^3−^ (mmol/L)	1.78 (1.57, 2.19)	1.91 (1.61, 2.22)	0.335	1.85 (1.59, 2.22)	1.85 (1.58, 2.15)	0.942
Ca^2+^ (mmol/L)	2.29 ± 0.17	2.34 ± 0.18	0.097	2.29 ± 0.19	2.32 ± 0.16	0.158
ipTH (pg/ml)	275.1 (165.45, 559.75)	396.5 (227.3, 562.6)	0.058	376.2 (208.13, 544.8)	318.95 (172.8, 564.45)	0.613
GLU (mmol/L)	6.29 (4.81, 8.07)	5.32 (4.55, 7.45)	0.055	6.53 (4.79, 9.1)	5.66 (4.65, 7.45)	0.070
HB (g/L)	112.14 ± 15.53	115.64 ± 13.00	0.116	113.42 ± 16.79	113.28 ± 13.15	0.948
ALB (g/L)	39.4 (37.5, 41.4)	40.2 (38.4, 42.2)	0.034	39.1 (37.35, 41.35)	40.15 (38.4, 42)	0.012
BMI (kg/m^2^)	23.52 (20.8, 25.72)	22.85 (20.36, 24.92)	0.286	23.76 (20.78, 26.10)	23.15 (20.44, 25.16)	0.191
MQSGA (score)	10 (10, 12)	10 (9, 11)	0.371	10 (10, 12)	10 (10, 11)	0.637
WC (cm)	0.87 ± 0.12	0.85 ± 0.12	0.189	0.89 ± 0.11	0.84 ± 0.12	0.004
**LTPA**						0.650
Inactive	/	/		57 (67.9%)	72 (64.3%)	
Active	/	/		27 (32.1%)	40 (35.7%)	
**NLTPA**			0.650			
Inactive	57 (44.2%)	27 (40.3%)		/	/	
Active	72 (55.8%)	40 (59.7%)		/	/	

ALB, albumin; BMI, body mass index; BUN, blood urea nitrogen; Ca^2+^, serum calcium; GLU, glucose; HB, hemoglobin; hs-CRP, high-sensitivity C-reactive protein; ipTH, parathyroid hormone; LTPA, leisure-time physical activity; MQSGA, modified quantitative subjective global assessment; NLTPA, nonleisure-time physical activity; P^3−^, serum phosphorus; TCH, total cholesterol; WC, waist circumference. Two independent-sample t-test, Mann–Whitney U-test, and chi-square test were used in normally distributed quantitative data, non-normally distributed quantitative data, and qualitative data, respectively.

**Table 3:** Relationship between physical activity types and sarcopenia risk.

**Physical activity**	**Incident cases/total (*n*)**	**RR^a^ (95%CI)**	**RR^b^ (95%CI)**	**RR^c^ (95%CI)**
**LTPA**				
Inactive	20/129	1	1	1
Active	9/67	0.866 (0.418, 1.797)	0.963 (0.477, 1.942)	0.964 (0.477, 1.951)
P		0.700	0.916	0.920
**NLTPA**				
Inactive	18/84	1	1	1
Active	11/112	0.458 (0.229, 0.918)	0.478 (0.272, 0.841)	0.449 (0.248, 0.814)
P		0.028	0.010	0.008

LTPA, leisure-time physical activity; NLTPA, nonleisure-time physical activity.

Model a: uncorrected. Model b: adjusted for covariates such as age, sex, BMI, ALB, MQSGA, WC, HGS, and SMM. Model c: adjusted for covariates such as age, sex, BMI, ALB, protopathy, nPAN, P, BUN, WC, HGS, and SMM.

## 4 Discussion

This prospective cohort study investigated the relationship between different types of physical activity and the risk of sarcopenia in patients undergoing MHD. After adjusting for factors such as age, sex, BMI, ALB, protopathy, nPAN, P, BUN, WC, HGS, and SMM, we found that nonleisure-time physical activity was significantly negatively associated with sarcopenia risk. These findings suggest that actively engaging in nonleisure-time physical activities can effectively reduce the risk of sarcopenia in MHD patients.

In our study, 14.8% of the participants developed sarcopenia over one year, a rate notably higher than the 5% to 10% typically observed in healthy older populations (42, 43). Previous studies have reported varying incidence rates of sarcopenia in MHD patients, ranging from 17.8% to 24.9% (44, 45), underscoring the elevated risk in this population. This increased susceptibility may be attributed to multiple factors, including reduced physical activity, selective muscle structural changes, decreased muscle strength, and significant muscle atrophy (46, 47). Additionally, MHD patients often experience protein–energy wasting, hormonal imbalances, disturbances in acid metabolism, and chronic inflammation (11), all of which contribute to the heightened risk of sarcopenia.

In our study, nonleisure-time physical activity emerged as the primary contributor to total daily physical activity. This aligns with a prospective survey of 150 MHD patients using the IPAQ, which also found that their physical activities were predominantly nonleisure-time activities (19). Unlike in Western countries, where leisure-time physical activities are more common (48, 49), Chinese MHD patients tend to rely more on nonleisure-time activities, such as household chores and daily mobility, as their primary sources of physical activity, which are part of their daily routine (18). Household responsibilities and daily tasks are often considered a core part of life in China. As a result, patients' physical activity is more likely to be derived from these tasks rather than from structured exercise or fitness activities aimed at maintaining health in this population. Notably, MHD patients who actively engaged in nonleisure-time physical activities had a 55.1% reduction in sarcopenia risk in our results. To the best of our knowledge, this is the first study to examine the relationship between sarcopenia and nonleisure-time physical activity specifically in MHD patients. Studies involving healthy individuals have demonstrated that nonleisure-time physical activities are beneficial for maintaining muscle mass and strength. Park et al. (50) found that nonleisure-time physical activities, such as regular farming, may help increase step counts in older individuals, thereby delaying the onset of sarcopenia. Moreover, Pan et al. (51) identified positive correlations between household physical activity and both muscle mass and strength, as well as between transport-related physical activity and muscle weight. These findings support the notion that both leisure-time and nonleisure-time physical activities can mitigate sarcopenia risk by enhancing muscle strength and mass. As few studies have specifically investigated the mechanisms by which nonleisure-time physical activity improves sarcopenia, its effects may be similar to the well-documented biological mechanisms of aerobic exercise in alleviating sarcopenia in MHD patients. These include stimulating muscle protein synthesis, reducing chronic inflammation (e.g., lowering TNF-α and IL-6 levels), improving mitochondrial function, enhancing hormonal regulation (e.g., increased IGF-1 and insulin sensitivity), and preserving neuromuscular function (52–54). Additionally, improved circulation from regular movement facilitates oxygen and nutrient delivery to muscles, counteracting atrophy (55, 56). Taken together, these mechanisms underscore the importance of exploring the potential impact of nonleisure-time physical activity on sarcopenia.

In contrast, while structured leisure-time physical activities have been shown to improve muscle mass and strength in both healthy individuals and patients with chronic diseases (57–62), our study did not find a significant relationship between leisure-time physical activity and sarcopenia risk. Our participants exhibited relatively low levels of leisure-time physical activity (231 METs/week in the sarcopenia group vs. 320 METs/week in the nonsarcopenia group). Although leisure-time physical activity was slightly higher in the nonsarcopenia group, the difference was not statistically significant, possibly due to the small sample size. Furthermore, implementing and maintaining structured leisure-time physical activities consistently in clinical settings remains challenging. Only 34.2% of patients in our study actively participated in leisure-time physical activities, and a previous large-scale survey indicated that over one-third1 of MHD patients had little or no participation in these activities, which is insufficient to confer health benefits (63). Additionally, in a study by Manfredini et al. (28), only 69% of patients on dialysis completed a 6-month leisure-time physical activity training program. From a practical and physiological perspective, nonleisure-time physical activities, as an integral part of daily life, are more frequent and regular, do not require additional time or resource allocation, and are easier to sustain over the long term, significantly increasing the overall energy expenditure of patients. In contrast, due to the lack of professional guidance, exercise-related knowledge, and limitations imposed by the disease itself, MHD patients often face challenges in maintaining participation in leisure-time physical activities, which may reduce their effectiveness in preventing sarcopenia (24, 64–66). Therefore, increasing nonleisure-time physical activities may serve as a more appropriate alternative for MHD patients. In clinical practice, while promoting structured physical activity programs, it is also important to evaluate and encourage nonleisure-time physical activities. Nephrologists and physical therapists should tailor physical activity intervention strategies to this population, encouraging increased daily activity through tasks such as carrying small objects, sweeping, window cleaning, and other household chores, as well as engaging in moderate-intensity physical activities related to transportation, such as walking (67). Individuals with chronic diseases, provided they have no contraindications, should maximize physical activity and minimize sedentary behavior to achieve optimal health benefits (68). This study follows the guidelines of the American College of Sports Medicine, setting 600 METs per week as the threshold for group classification (41). We recommend that patients engage in at least 600 METs of nonleisure-time physical activity per week to confer protective effects against sarcopenia. However, since evidence on nonleisure-time physical activity remains relatively limited, future research should explore the minimum METs/week threshold for sarcopenia prevention and the potential biological mechanisms linking nonleisure-time physical activity and sarcopenia, providing stronger evidence to support its feasibility and ultimately benefiting more patients with low levels of physical activity.

This study has several limitations. First, the IPAQ is a self-reported and recall-based questionnaire to evaluate physical activity; however, it can distinguish between leisure and nonleisure-time physical activities compared with accelerometers or pedometers. Second, the data were collected from a single center with a limited number of cases and a short follow-up period, which may not adequately represent the entire population undergoing MHD. Therefore, extensive multicenter studies with larger sample sizes and longer follow-up periods are warranted to further investigate the relationship between physical activity types and sarcopenia risk in MHD patients. Finally, while our study did not explore the impact of dietary composition on sarcopenia, future research should further investigate the role of nutritional components and dietary patterns in sarcopenia prevention and management in MHD patients to enhance clinical guidance.

## 5 Conclusion

The incidence of sarcopenia is notably high among patients undergoing MHD. Most of these patients engage in nonleisure-time physical activities. Actively participating in such activities can significantly reduce the risk of sarcopenia. Furthermore, nonleisure-time physical activities are often easier to implement and more readily accepted by MHD patients, making them an effective strategy to enhance the physical fitness and overall health of individuals undergoing MHD.

## Data Availability

The raw data supporting the conclusions of this article will be made available by the authors, without undue reservation.
